# Exploring salivary microbiota in AIDS patients with different periodontal statuses using 454 GS-FLX Titanium pyrosequencing

**DOI:** 10.3389/fcimb.2015.00055

**Published:** 2015-07-02

**Authors:** Fang Zhang, Shenghua He, Jieqi Jin, Guangyan Dong, Hongkun Wu

**Affiliations:** ^1^State Key Laboratory of Oral Diseases, West China College of Stomatology, Sichuan UniversityChengdu, China; ^2^Public Health Clinical Center of ChengduChengdu, China; ^3^Department of Geriatric Dentistry, West China College of Stomatology, Sichuan UniversityChengdu, China

**Keywords:** acquired immunodeficiency syndrome, opportunistic infections, periodontal diseases, microbiota, diversity

## Abstract

Patients with acquired immunodeficiency syndrome (AIDS) are at high risk of opportunistic infections. Oral manifestations have been associated with the level of immunosuppression, these include periodontal diseases, and understanding the microbial populations in the oral cavity is crucial for clinical management. The aim of this study was to examine the salivary bacterial diversity in patients newly admitted to the AIDS ward of the Public Health Clinical Center (China). Saliva samples were collected from 15 patients with AIDS who were randomly recruited between December 2013 and March 2014. Extracted DNA was used as template to amplify bacterial 16S rRNA. Sequencing of the amplicon library was performed using a 454 GS-FLX Titanium sequencing platform. Reads were optimized and clustered into operational taxonomic units for further analysis. A total of 10 bacterial phyla (106 genera) were detected. *Firmicutes*, *Bacteroidetes*, and *Proteobacteria* were preponderant in the salivary microbiota in AIDS patients. The pathogen, *Capnocytophaga* sp., and others not considered pathogenic such as *Neisseria elongata*, *Streptococcus mitis*, and *Mycoplasma salivarium* but which may be opportunistic infective agents were detected. *Dialister pneumosintes*, *Eubacterium infirmum*, *Rothia mucilaginosa*, and *Treponema parvum* were preponderant in AIDS patients with periodontitis. Patients with necrotic periodontitis had a distinct salivary bacterial profile from those with chronic periodontitis. This is the first study using advanced sequencing techniques focused on hospitalized AIDS patients showing the diversity of their salivary microbiota.

## Introduction

Acquired immunodeficiency syndrome (AIDS) is the advanced stage of human immunodeficiency virus (HIV) infection. The progressively weakened immune system makes the host vulnerable to series of selected conditions and opportunistic infections. In 2012, about 1.6 million adults and children died of AIDS worldwide (UNAIDS, 2013). As prominent features of HIV infection and AIDS, oral manifestations have been associated with the level of immunosuppression, and are considered as an indication of exacerbation and progression (Greenspan et al., 2004). Among these oral manifestations, various types of periodontal diseases are regarded as serious complications of HIV infection and have important diagnostic and prognostic values (Coogan et al., 2005). HIV-associated periodontal diseases include specific forms of gingivitis and periodontitis and include linear gingival erythema, necrotic gingivitis and necrotic periodontitis (UNAIDS, 2013). With the clinical implementation of antiretroviral therapy/highly active antiretroviral therapy, these specific periodontal diseases may be less common, but still occur in part because of increased life expectancy (Ryder et al., 2012). A previous study compared the microbiota between healthy controls and patients with HIV, and showed that patients with HIV had an increased oral colonization by *Micrococcus* sp. (a normal commensal of the skin) (Hegde et al., 2014). Another study showed that *Entamoeba gingivalis*, an oral commensal, had pathogenic potentials in immunocompromised individuals (Cembranelli et al., 2013). Microbiological shift, behavior and immune function of the host all contribute to the etiology of infectious diseases in these patients (Marsh, 2003).

Periodontitis is a polymicrobial infection mediated by the immune response of the host and oral microbes (Perez-Chaparro et al., 2014). The oral environment contains both commensal and pathogenic microbes, with approximately 600 prokaryote species documented by the Human Oral Microbiome Database (HOMD) (Dewhirst et al., 2010). New technologies continue to facilitate the better understanding of the microbial etiology of periodontitis and how the patient's systemic status interacts with oral health and disease process. Previous studies showed that fungi (such as *Candida* sp.) and a number of bacteria were involved in oral diseases of AIDS patients (Gonçalves et al., 2009; Mukherjee et al., 2014). However, these previous studies mostly used selective culture media or PCR to identify the microorganisms, which leads to a number of microorganisms being missed, or did not assess the differences in microorganisms between different degree of periodontal health in patients with AIDS. Therefore, the oral microbiota in AIDS patients and its function in the pathogenesis of periodontal disease still need further investigation.

Simultaneous progress in sequencing technology, availability of genome sequences and bioinformatics has allowed for the development of next-generation sequencing based on pyrosequencing (Gilles et al., 2011). Sequencers such as the 454 GS-FLX Titanium pyrosequencing system (Roche Diagnostics, Basel, Switzerland) provide about 1,000,000 high-quality sequences in a single 10-h run, and can be used to shotgun libraries of genomes (Gilles et al., 2011).

The aim of this study was to explore the salivary bacterial diversity of oral microbiota in AIDS patients with different levels of periodontal health using a 454 GS-FLX Titanium pyrosequencing system to sequence the 16S rRNA to identify microbial species. Our data will help to define the overall structure of salivary microbiota in AIDS patients and to understand the microbial changes in oral cavity during severe immunosuppression, which will be beneficial in the clinical treatment for periodontal diseases associated with AIDS.

## Materials and methods

### Study population

Fifteen patients with AIDS (Table 1) were randomly recruited between December 2013 and March 2014 from patients newly admitted to the AIDS ward of the Public Health Clinic Center (PHCC), Chengdu, China. Information regarding demographic features, general health, and HIV infection history were obtained from anamnesis questionnaire and patients' medical records.

**Table 1:** **Demographic and clinical data of the patients**.

**Patient ID**	**Age (years)**	**Sex**	**CD4**	**CD4/CD8 ratio**	**HIV viral load**
106367	48	M	11	0.01	9.49E+05
106476	40	F	28	0.11	2.46E+06
105683	51	F	18	0.04	9.58E+06
105971	41	M	10	0.06	6.23E+05
105768	56	M	30	0.03	6.21E+06
106074	47	M	34	0.02	5.52E+05
105675	55	M	13	0.04	7.62E+05
106495	46	M	44	0.35	6.26E+04
104605	50	M	33	0.09	3.98E+06
105166	51	M	51	0.14	5.36E+06
103135	44	M	74	0.17	7.14E+05
104664	49	M	16	0.03	8.21E+06
103684	53	M	46	0.02	1.75E+06
105305	42	M	11	0.04	2.24E+07

HIV infection was diagnosed in the presence of: (1) any stage 4 condition with confirmed HIV infection; (2) immunological diagnosis; or (3) first-ever documented CD4 count less than 200 per mm^3^ or %CD4+ <15 (WHO, 2007).

Inclusion criteria were: (1) newly admitted patients with an AIDS diagnosis (UNAIDS, 2013); (2) over 22 years of age; and (3) at least 20 teeth. Two experienced dentists performed the full-mouth examination of all patients to determine their oral health status. Then, patients were allocated to one of three groups according to periodontal status: periodontal health, gingivitis, and periodontitis, which can be subcategorized into chronic periodontitis and AIDS-related necrotic periodontitis.

Exclusion criteria were: (1) pregnancy; (2) nursing; (3) diabetes mellitus; (4) hypertension; (5) autoimmune diseases; (6) use of antibiotics within 3 months before or during admission; (7) oral tumor; or (8) bacterial or viral infections with clinical symptoms (obvious pseudomembranous or erythematosus Candidiasis, stomatitis, herpes simplex, acute posterior ganglionitis, lingual margin hairy leukoplakia, Kaposi's sarcoma, and aphtha).

The Ethical Committee of the West China Hospital of Stomatology, Sichuan University and the Research Department of PHCC approved the study protocol (WCHSIRB-D-2-14-052). Written informed consent was obtained from each participant.

### Definitions of periodontal status

Among included participants, three were in the periodontal health group, five were diagnosed with gingivitis, and seven suffered from periodontitis (including two with AIDS-related necrotizing periodontitis).

Periodontal health was defined as clinically healthy gingiva without bleeding on probing, no attachment loss, probing depth ≤3 mm, and no radiographic evidence of bone loss. In the gingivitis group gingiva presented red to bluish red edematous appearance with swollen inter-dental papillae and increased tendency of bleeding. Each patient had at least four sites with gingivitis according to the following criteria: gingival index >0, probing depth <3 mm, and no attachment loss (Feller and Lemmer, 2008). Subjects with chronic periodontitis had at least four sites meeting the following criteria: gingival index >0, probing depth >5 mm, and attachment loss >5 mm (Feller and Lemmer, 2008). AIDS-related necrotic periodontitis group was diagnosed by necrotic appearance of periodontal attachment, gingival bleeding and pain (Feller and Lemmer, 2008).

### Saliva sampling and processing

Patients retained saliva in the mouth, allowing collection of 5 ml of unstimulated saliva from each patient at least 2 h after the last meal using a 15 ml centrifuge tube (Corning Inc., Corning, NY, USA) that was slightly stuck to the inner mucosa of the underlip. Samples not severely contaminated with blood were immediately transported on dry ice to the laboratory in PHCC. Saliva samples were centrifuged at 2600 g for 10 min to discard large debris and eukaryotic cells. An aliquot of 1.5 ml of the supernatant from each sample was then centrifuged again at 14,000 g for 5 min and the pellet was collected for DNA extraction (Tian et al., 2010).

A MasterPure™ DNA purification kit (Epicentre, Madison, WI, USA) was used to extract the total genomic DNA of bacteria from all samples. The quality and quantity of the products were measured using an UV spectrophotometer (NanoVue™, GE Healthcare, Waukesha, WI, USA) at 260 and 280 nm. All genomic DNA samples were stored at −80°C before further analysis.

### PCR and pyrosequencing

PCR amplification of the bacterial 16S rRNA gene hypervariable V3–V5 region was performed using the universal bacterial primers 347F (5′-GGA GGC AGC AGT RRG GAA T-3′) and 803R (5′-CTA CCR GGG TAT CTA ATC C-3′) (Nossa et al., 2010), incorporating the 454 universal adapters and multiplex identifier at the 5′ end of the reverse primer. The PCR reactions were carried out by 2 min initial denaturation at 95°C, 25 cycles of denaturation at 95°C (30 s), annealing at 60°C (30 s), elongation at 72°C (30 s), and one final extension at 72°C for 5 min. Products were purified with the AMPure XP PCR purification Kit (Beckman Coulter, Brea, CA, USA) to remove any primer dimers. PCR products were qualified and quantified using LabChip GX (Calipier Life Sciences, A PerkinElmer company, Waltham, MA, USA). An amplicon library was built and applied to 454 pyrosequencing according to the manufacturer's recommendations. Pyrosequencing was performed unidirectionally from the 347F primer end on a 454 GS-FLX System platform (Roche Diagnostics, Basel, Switzerland) in a single full-plate run.

### Sequence and statistical analysis

The V3–V5 region in the hypervariable region of 16S rDNA was sequenced. Raw pyrosequencing results were filtered according to primer sequences using a combination of tools from Mothur (version 1.31.2; http://www.mothur.org). Unique reads were extracted as follows: (1) All reads were assigned to the corresponding samples after mapping with barcode and primer sequences. Mismatches between reads and barcode were at most 1 bp and unsuitable reads were excluded. (2) Low quality reads would be produced in the processing of 454 sequencing. Average quality of raw reads was assessed using the Mothur software with a threshold of 25. Reads containing base N, containing homopolymers longer than seven nucleotides (such as AAAAAAAA), and shorter than 200 bp or longer than 1000 bp were excluded. (3) Read redundancy was filtered using the Mothur software to select Unique Reads sequences, which represented a group of identical tag sequences of variable amounts. (4) All reads were aligned with reference database SILVA alignment (v102) using NAST algorithm, and assigned to target region. Other non-targeted reads were excluded. (5) Preliminary clustering was performed using the Mothur software for Unique Reads with threshold of 1 mismatch per 100 bp, which meant that low-abundant sequences were added into high-abundant sequences with a difference less than 1%. It was supposed that low-abundant sequences were derived from high-abundant sequences. So this step was mainly used to reduce the number of wrong OTU. (6) Chimera sequences were identified by UCHIME (v4.2, http://drive5.com/uchime) algorithm and such sequences were excluded. (7) Species annotation was performed using classifier software (based on Naïve Bayesian Classifier) involved in the Mothur software based on the RDP database (16S rRNA training set 9, http://www.mothur.org/wiki/RDP_reference_files) (Schloss et al., 2009), with the smallest bootstrap as 80%. Reads were excluded if the reads were annotated as chloroplast or mitochondria.

OTUs that reached 97% similarity level were used for alpha-diversity using Mothur (Chao et al., 1992), richness (Chao and Bunge, 2002) and rarefaction curves using the R 2.15.3 software (Schloss et al., 2009). Venn diagrams were created using the R software (Chen and Boutros, 2011). Beta diversity analysis showed the species diversity among different samples. By analyzing the level of different species in specific samples, Beta-diversity was calculated using QIIME (version 1.50, http://qiime.org/index.html) and rendered by the R software. To explore the species diversity among samples, Principal coordinate analysis (PCoA) was performed according to the distance matrices calculated by QIIME (Crawford et al., 2009). A close distance between two samples meant similar species composition between these two samples. The results were achieved by 100 calculations for random selection and dark dots were the final results of 100 calculations with lighter area as the results of each calculation. If the reproducibility of the sample was good, the lighter area range was small while poor reproducibility resulted in a larger lighter area range. In each sample with or without weighing the abundance of species respectively, 75% Reads were randomly selected for variance calculation, and final statistical results and PCoA figure was achieved after 100 iterative computation. Cluster analysis was performed by R using UPGMA (Unweighted Pair Group Method with Arithmetic mean) analysis. The rank-sum test or Kruskal-Wallis test was used to analyze the differences in diversity indices and bacterial relative abundance. Heatmap analysis was performed according to the relative abundance of each species in each sample. The top 30 species with the highest abundance were selected and a heatmap was made using pheatmap software in (R v2.15.3), with correlation distance algorithm and complete clustering method. All statistical analysis was performed using SPSS 19.0 for Windows (IBM, Armonk, NY, USA).

## Results

### Results metrics

**Figure 1:**
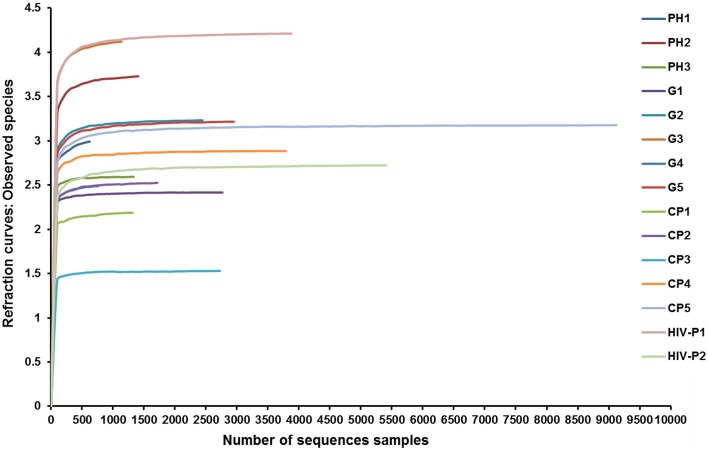
**Rarefaction curves of observed species**. Rarefaction curves comparing the number of reads with the number of observed species found in the DNA from the saliva of AIDS patients with different periodontal statuses. PH, Periodontal Health; G, Gingivitis; P, Periodontitis.

The total number of reads and the number of effective reads obtained from the original FASTA file by parallel high-throughput pyrosequencing of each saliva sample before and after quality control procedures are shown in Table 2. Mean length of the reads was 242 base pairs. A total number of 81,255 unique reads were classified as bacteria and subsequently used for analysis of diversity and relative abundance. The mean number of reads per patient was 4896 ± 3614 (range: 1191–14,862). The mean number of reads that passed quality control was 2759 ± 2207 (range: 624–9123), for an effective read ratio of 55.0 ± 6.3%. The mean number of OTUs per participant was 98 ± 58. The rarefaction curves indicate that these unique reads were adequate for further analyses for most samples since increasing the number of reads beyond that value had minimal contribution to the number of OTUs (Figure 1).

**Table 2:** **Number of reads for each sample in the original data sets and after filtering and classification**.

**Sample**	**Original number of reads**	**Effective reads pass quality control**	**Effective reads ratio (%)**	**OTU number**
PH1	1191	624	52.39	67
PH2	2398	1409	58.76	123
PH3	2643	1334	50.47	33
G1	5225	2766	52.94	36
G2	3529	2446	69.31	106
G3	2425	1137	46.89	131
G4	1584	762	48.11	48
G5	5189	2951	56.87	126
P1	2441	1316	53.91	47
P2	3170	1715	54.10	62
P3	6084	2725	44.79	34
P4	6140	3788	61.69	89
P5	14862	9123	61.38	197
P6	6778	3877	57.20	204
P7	9774	5413	55.38	162

### Relative abundances

Bacterial phyla are presented in Table 3. In AIDS patients with periodontal health, *Firmicutes* (37.4%) and *Bacteroidetes* (32.7%) predominated, followed by *Fusobacteria* (9.1%) and *Actinobacteria* (8.3%). In the gingivitis group, *Firmicutes* (38.8%) and *Bacteroidetes* (32.8%) predominated, followed by *Proteobacteria* (14.1%) and *Actinobacteria* (9.0%). In AIDS patients with periodontitis, *Firmicutes* was the most prevalent phylum (53.9%), followed by *Bacteroidetes* (22.5%). *Tenericutes* was the only phylum that was not present in AIDS patients with periodontal health. SR1 and *Deinococcus-Thermus* were only identified in patients with gingivitis. *Prevotella*, *Streptococcus*, *Veillonella*, *Actinomyces*, and *Fusobacterium* accounted for 73.6% of 50 genera identified in patients with periodontal health. Bacterial composition distinguished the gingivitis group from the healthy group. Nine genera (*Streptococcus*, *Prevotella*, *Capnocytophaga*, *Veillonella*, *Granulicatella*, *Neisseria*, and *Actinomyces*) accounted for 78.2% of 74 genera found in patients with gingivitis. As to patients with periodontitis, only four genera accounted for 61.7% of all 106 genera. The relative abundance of each genus was compared between the groups. The presence of *Porphyromonas* sp., *Treponema* sp., and *Eubacterium* sp. was significantly higher in the periodontitis group compared with the other groups (*P* < 0.05).

**Table 3:** **Distribution of salivary bacteria at the phylum level in each group**.

**Phylum**	**Relative distribution %**		
	**Periodontal health**	**Gingivitis**	**Periodontitis**
*Actinobacteria*	8.3	9.0	7.1
*Bacteroidetes*	32.7	32.8	22.5
*Chloroflexi*	0	0	0
*Deinococcus-Thermus*	0	0.2	0
*Fusobacteria*	9.1	2.4	3.0
*Planctomycetes*	0	0	0
*Proteobacteria*	10.0	14.1	9.2
*Spirochaetes*	2.3	1.6	3.0
*Synergistetes*	0	0	0
*Tenericutes*	0	0.1	0.2
*Firmicutes*	37.4	38.8	53.9
Other	0.1	0.9	1.0
*SR1*	0	0.9	0
*TM7*	0	0	0

The relative abundance of *Desulfobulbus* was significantly higher in the gingivitis group compared with periodontitis group (*P* = 0.019), while *Streptococcus* was lower in the gingivitis group compared with the periodontitis group (*P* = 0.030). *Johnsonella* had a greater abundance in the periodontal health group (*P* = 0.032, respectively).

At the species level, *Veillonella atypica* (*P* = 0.013) showed a higher abundance in the periodontal health group compared with the gingivitis group. However, *Selemonas infelix* (*P* = 0.026) was lower in the periodontal group compared with the gingivitis group. *Johnsonella ignava* (*P* = 0.032) and *Treponema leuthinolyticum* (*P* = 0.032) were with higher abundance in the periodontal health group compared with the periodontitis group, while *Dialister pneumosintes* (*P* = 0.035), *Eubacterium infirmum* (*P* = 0.049), *Rothia mucilaginosa* (*P* = 0.021) and *Treponema parvum* (*P* = 0.045) were with significantly greater abundance in the periodontitis group.

### OTU analysis

Alpha-diversity is a measure of a species' abundance in an ecosystem. The indices of diversity and richness are shown in Table 4. The comparisons of alpha-diversity indices of the saliva microbiota were not significantly different between the three groups at a 3% cutoff level.

**Table 4:** **Alpha diversity indices in each group of AIDS patients with different periodontal statuses**.

	**OTU**	**ACE**	**Chao**	**Shannon**	**Simpson**
Periodontal health	145	113.17 ± 50.81	122.34 ± 51.67	3.10 ± 0.58	0.083 ± 0.026
Gingivitis	224	131.23 ± 43.05	131.80 ± 40.90	3.09 ± 0.69	0.091 ± 0.047
Periodontitis	416	227.70 ± 122.83	175.44 ± 94.16	2.74 ± 0.84	0.179 ± 0.113

*The operational taxonomic units (OTUs) were defined at a 3% cutoff level*.

*Richness estimators (ACE and Chao), diversity indices (Shannon and Simpson) were calculated using the Mothur software*.

**Figure 2:**
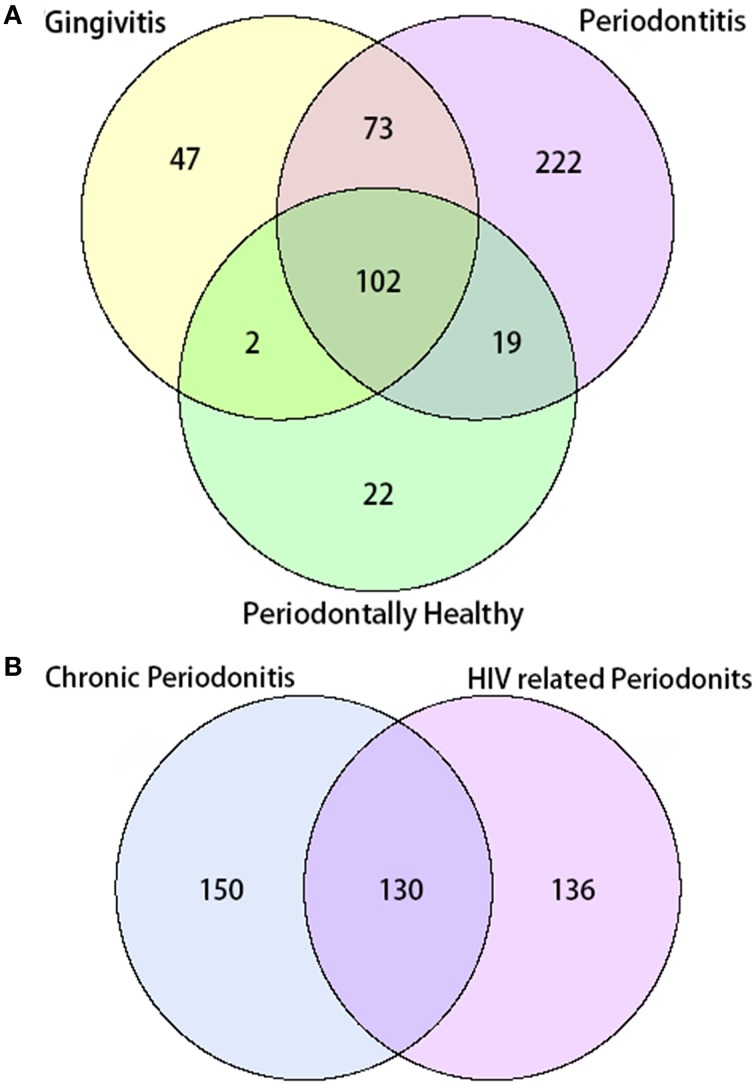
**Venn diagram of the number of species shared/distinct within (A) all three groups and (B) subgroups with chronic and AIDS-related periodontitis**. The overlapping area represents the set of bacteria shared between groups, while the single-layer part represents the number of bacteria distinctly found in a certain group.

In total, 487 bacterial species were present in all three groups. There were 102 species shared between all three groups, while 22 species were uniquely observed in the periodontal health group. Additionally, there were 47 unique species observed in the gingivitis group, and 222 unique species in the periodontal group. A total number of 130 species were shared by the chronic periodontitis group and the HIV-related necrotic periodontitis group. However, they also had 150 and 136 exclusive species, respectively (Figure 2).

**Figure 3:**
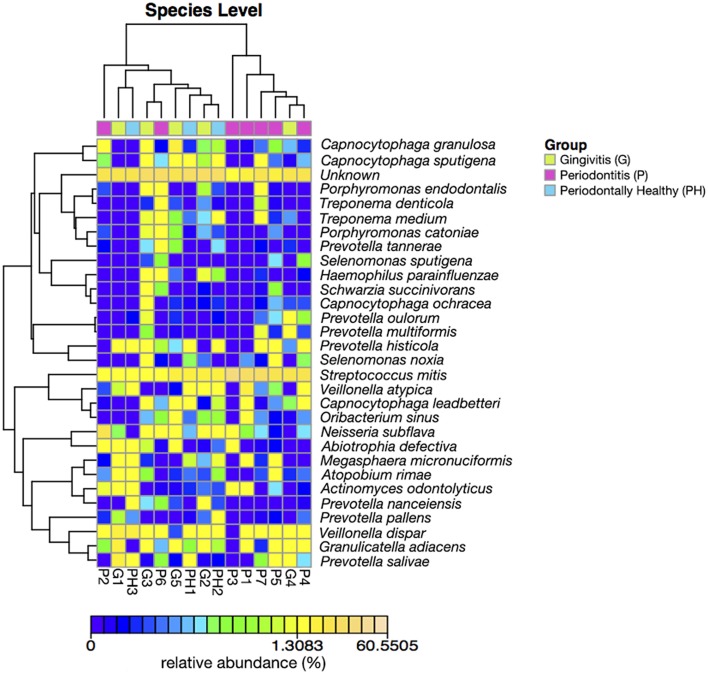
**Heatmap of relative abundance at species level of salivary bacterial profile in AIDS patients with different periodontal statuses**.

**Figure 4:**
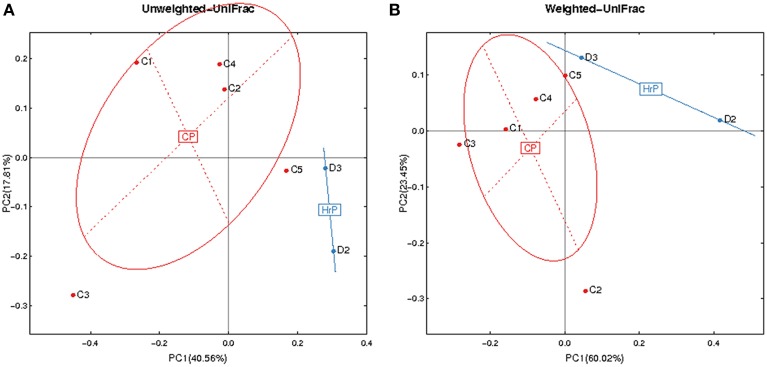
**Principal Coordinates Analysis (PCoA) based on relative abundance of OTUs identified in the saliva of AIDS patients. (A)** Unweighted. **(B)** Weighted. C1-C5, ID of patients with chronic periodontitis (in red). D2-D3, ID of patients with AIDS-related necrotizing periodontitis (in blue). CP, chronic periodontitis; HrP, HIV/AIDS-related necrotizing periodontitis.

Figures 3, 4 show the analysis results for species diversity among samples analyzed by PCoA. A heatmap was used to demonstrate the profile of salivary microbiota in AIDS patients (Figure 3): 71.4% of all samples in the periodontitis group and one sample from the gingivitis group were clustered in one tree, while all the other samples were clustered in the other tree, which were mainly samples from the periodontal health and gingivitis groups. Salivary microbiota in AIDS patients showed discrepancy between groups with and without periodontitis.

Given the particularity of HIV-related necrotic periodontitis, we used a PCoA plot based on unweighted and weighted UniFrac distance metrics to demonstrate the differences in salivary microbiota between necrotic and chronic periodontal disease (Figure 4).

## Discussion

AIDS patients are in a long-term compromised immune state, and the resulting effect on oral microbiota and its relationship with chronic oral infectious diseases are not fully understood. Due to the increased availability and performance of high throughput DNA sequencing platforms, pyrosequencing was used to directly sequence 16S rRNA to survey the salivary microbiota of AIDS patients.

The present study is the first to use a high throughput DNA sequencing technology to assess the differences in AIDS patients with or without periodontal diseases. 10 bacterial phyla (106 genera) were detected. *Firmicutes*, *Bacteroidetes*, and *Proteobacteria* were preponderant in the salivary microbiota in AIDS patients. Potential opportunistic infective agents, such as *Neisseria elongatas* and *Mycoplasma salivarium* were detected as well as pathogenic *Capnocytophaga* sp. *D. pneumosintes*, *E. infirmum*, *R. mucilaginosa*, and *T. parvum* were preponderant in AIDS patients with periodontitis. Patients with necrotic periodontitis had a different salivary bacterial profile (cluster analysis, Venn diagram) from those with chronic periodontitis. These results are supported by increasing evidence showing that associations exist between the quantity of salivary pathogenic bacteria and the severity of periodontal diseases (Monteiro et al., 2014).

Previous studies analyzed AIDS patients' saliva using conventional techniques (selective media and PCR techniques) (Hegde et al., 2014; Mukherjee et al., 2014). Using the 454 pyrosequencing technology, the present study was the first to identify the phylotypes at a 3% cutoff level to assess the bacteria present in the oral cavity of AIDS subjects in relation with their periodontal health. For 454 sequencing analysis the default cut-off is 3%, with a suggested 95% similarity needed to define genus and 97% for species (Tindall et al., 2010), A total of 487 OTUs were obtained while previous studies obtained only 111–377 OTUs in different parts of the oral cavity (Zaura et al., 2009; Diaz et al., 2012). However, those previous studies were concerned with HIV negative subjects. Species of the salivary microbiota might be increased under immunocompromised condition, but we did not compare these results with healthy controls to evaluate this. Among these OTUs, only 5% of all the sequences represented the majority of the salivary microbiota, indicating that a large number of species are at really low levels.

Alterations in the oral microbial communities in AIDS patients is of great importance due to their close relationship with oral diseases and the high risk of infection caused by immunodeficiency (Bruno et al., 2003). Previous studies have also investigated the microbiota in the oral cavity of patients with HIV or AIDS. A previous study using oral rinse as the sampling method showed that *Prevotella*, *Streptococcus*, and *Rothia* were the most common genus in HIV-positive subjects (Mukherjee et al., 2014). In our study, *Firmicutes* (*Streptococcus* and *Veillonella*) and *Bacteroidetes* (*Prevotella*) were the predominant phyla in the saliva of AIDS patients. However, the presence of *Rothia* sp. was relatively low. This discrepancy might be due to the status of patients' immune function and sampling methodology, as well as the specific population being studied. A study also using the oral rinsing sampling method found that when compared with healthy controls there was a shift in oral microflora in HIV infected patients with a reduction in the isolation of *Viridans streptococci* and *S. pneumoniae*, but an increase in *Micrococcus* sp. (Hegde et al., 2014). While a comparison of those patients with HIV that had been treated with antiretroviral therapy, those who were antiretroviral naïve, and healthy controls using tongue samples and PCR/microarray methods showed that potential pathogenic *Veillonella, Prevotella, Megasphaera*, and *Campylobacter* were increased in antiretroviral naïve HIV infection while commensal *Streptococcus* and *Veillonella* species and *Neisseria flavescens* were lower (Dang et al., 2012). In the patients receiving antiretroviral therapy lower relative proportions of *Lachnospiraceae* and *Neisseria* appeared to be counterbalanced by higher relative proportions of other genera, higher *Megasphaera* and *Streptococcus* species. Suggesting that administration of antiretroviral therapy may lead to alterations in the phylogenetic profile of the oral microbiota that are fundamentally distinct from the changes associated with untreated HIV infection (Dang et al., 2012).

Alpha-diversity analysis did not show any significant difference in the salivary microbiota of AIDS patients with different periodontal conditions. However, cluster analysis demonstrated that the distribution of *V. atypica*, *D. pneumosintes*, *E. infirmum*, *J. ignava*, *R. mucilaginosa*, *Treponema lecithinolyticum*, and *T. parvum* were significantly different in AIDS patients with healthy peridontium compared with those with gingivitis. However, classic periodontal pathogens were not significantly different between these two groups. These results are supported by previous suggestions that uncommon species might affect the process of periodontitis in AIDS patients (Hegde et al., 2014). Forty-five unique OTUs that were found in HIV-related necrotic periodontitis, suggesting that they might play roles in the pathogenesis of necrotic lesion, but further analysis in future studies will be needed to test this. Previous studies suggested that there might be no difference in subgingival microbiota between common periodontal diseases and necrotic periodontitis in HIV-positive patients (Murray et al., 1991). However, in the present study, we found 136 exclusive species in HIV-related periodontitis. PCoA plots also provided evidence to support a distinction in microbial profiles in HIV-related necrotic periodontitis. Although the incidence of necrotic periodontitis usually decreases due to highly active antiretroviral therapy, additional research is required because of its distinctive, destructive and irreversible features, and because of its potential role in indicating progression of the HIV infection.

In a previous study of necrotizing periodontal diseases in HIV infected patients samples from subgingival biofilms were collected from necrotizing lesions of six patients (Ramos et al., 2012). The species detected with high prevalence and/or counts included *Treponema denticola, Eikenella corrodens, D. pneumosintes, Enterococcus faecalis, Streptococcus intermedius, Aggregatibacter actinomycetemcomitans*, and *Campylobacter rectus* (Ramos et al., 2012). In order to investigate specific bacteria involved in HIV-related necrotic periodontal lesion in our study, we reviewed the species uniquely detected in necrotic periodontal patients. *Capnocytophaga* sp. is a common genus that can be isolated from periodontal pockets, periapical abscess and periodontal abscess (McGuire and Nunn, 1996). In addition, it was reported to cause septicemia, pulmonary abscesses, endocarditis and meningitis (Desai et al., 2007). *D. pneumosintes* is a relatively new species related to periodontitis (Ghayoumi et al., 2002). It can be isolated from clinical samples of deep periodontal pockets and pulp infections, and is involved in brain abscesses (Rousee et al., 2002). However, its relationship with destructive periodontal lesion is still not fully understood (Contreras et al., 2000). *T. parvum* and *Treponema putidum* are mainly seen in periodontitis and acute necrotic, ulcerative gingivitis (Wyss et al., 2004). *T. lecithinolyticum* was identified in our study. It was related to periodontitis, and is more present in rapid aggressive periodontitis than in chronic periodontitis (Wyss et al., 1999). Our findings indicate that they might also be involved in HIV-related necrotic periodontal lesions.

The oral cavity is a complex microbial ecological environment with a myriad of microorganisms that have a close relationship with oral health and diseases, and even have effects on the health of other body parts (Schmidt et al., 2014). Pathogens that might cause oral and systemic infectious diseases were detected in this study. *N. elongata* is a member of normal flora in oral cavity, but it may cause endocarditis and osteomyelitits (Avila et al., 2009). *Streptococcus mitis* can transfer possible virulence factors to other bacterial pathogens such as *Streptococcus pneumoniae* (Bensing et al., 2001). *Capnocytophaga* sp. is a well-recognized commensal and opportunistic pathogen; it is involved in the pathogenesis of periodontal diseases (Jolivet-Gougeon et al., 2007), and its pathogenicity is effected by the immune function of the host (Meyer et al., 2008), causing septicemia is in immunocompromised patients (Pokroy-Shapira et al., 2012). *Mycoplasma* sp. is also found in the normal flora in oral cavity (Watanabe et al., 1986), and certain species such as *M. salivarium* were reported to cause serious infections in HIV-positive patients (Chattin-Kacouris et al., 2002). *Actinomyces odontolyticus* might cause pulmonary actinomycosis, septicemia, and pulmonary abscesses (Rajesh et al., 2007). Even though most species are not pathogenic, certain members of the *Corynebacterium* genus are important pathogen in immunocompromised patients (Dinic et al., 2013).

This study has some limitations. Due to the small sample size, it is important to be aware that the findings are a preliminary indication of the impact of AIDS on the oral microbiota and their relationship to periodontal status. The microbial profile of an individual can be difficult to define as there are transient species whose prevalence can vary depending on time of sampling, diet, oral hygiene, and numerous other factors. We selected saliva samples as the method for analysis; however, directly sampling from subgingival plaques may have provided a more direct link to periodontal status, but could increase the risk of opportunistic infections in these patients. Thus, this study should be regarded as the starting point for more in-depth analysis including the inclusion of a healthy control population to fully evaluate the microbiota of AIDS patients and the relationship with severity of periodontitis.

In conclusion, AIDS patients with different periodontal statuses had different saliva microbial profiles. Particular species might be involved in the development of AIDS-related periodontitis. Myriads of commensal and opportunistic pathogens were identified, and they might cause severe and life-threatening complications in AIDS patients. Therefore, the microbial species involved in the pathogenesis of AIDS-related periodontitis patients require more extensive and comprehensive investigation using well-designed longitudinal studies. Oral healthcare should be emphasized in patients with AIDS. Oral preventive and therapeutic services should be provided to reduce the risk of serious infections in HIV-positive and AIDS patients. The results of the present study identified microorganisms that could be specifically targeted for the prevention of periodontal diseases in AIDS patients.

## Author contributions

FZ and SH carried out the data collection and analysis, wrote the manuscript. JJ and GD participated in data collection and help to perform the statistical analysis. HW conceived of the study, and participated in its design and coordination and provided the critical revision. All authors read and approved the final manuscript.

### Conflict of interest statement

The authors declare that the research was conducted in the absence of any commercial or financial relationships that could be construed as a potential conflict of interest.
